# What role does the Notch signaling pathway play in exercise-related metabolic and neurological adaptations? A molecular-to-systems perspective

**DOI:** 10.3389/fphys.2026.1772055

**Published:** 2026-06-08

**Authors:** Lin Li, Jianda Kong, Xuewen Tian

**Affiliations:** 1 Department of Sports Science Research Institute, Shandong Sport University, Jinan, China; 2 Department of Physical Education, Qufu Normal University, Jining, China

**Keywords:** exercise, metabolic homeostasis, neurogenesis, neuroplasticity, Notch signaling, oxidative stress

## Abstract

The Notch signaling pathway is a highly conserved cell–cell communication system that plays central roles in stem-cell maintenance, tissue homeostasis, cell-fate determination, and metabolic regulation. Because exercise induces coordinated adaptations across the nervous, muscular, cardiovascular, and metabolic systems, Notch signaling has emerged as a potential mediator of exercise-associated plasticity. However, whether exercise directly activates or suppresses Notch signaling in a causal, tissue-specific, and intensity-dependent manner remains unresolved. In this narrative review, we synthesize evidence on canonical and non-canonical Notch signaling, its functions in neural and metabolic regulation, and its potential intersections with exercise-related neurogenesis, muscle remodeling, redox balance, and metabolite signaling. We contend that the current evidence is best understood within a context-dependent framework rather than through a universal model of exercise-induced Notch activation. In particular, categories such as “moderate” and “high-intensity” exercise should be interpreted as individualized physiological domains defined relative to markers including lactate and ventilatory thresholds, cardiorespiratory reserve, and baseline fitness. We further propose that exercise-derived metabolites, including lactate, ketone bodies, and shifts in cellular NAD+/AMP status, may modulate Notch-related signaling indirectly or in a cell-type-specific manner; however, these interactions should currently be regarded as hypothesis-generating rather than established linear pathways. Across tissues, the strongest mechanistic evidence pertains to Notch biology in neural stem cells, synaptic plasticity-associated signaling, and skeletal-muscle stem-cell regulation, whereas direct human exercise studies assessing Notch pathway activation remain scarce. We therefore propose a context-dependent working model in which Notch acts as a potential integrator of exercise-responsive neural and metabolic cues, while also emphasizing major limitations, conflicting findings, and the safety concerns associated with systemic pharmacological modulation of this pathway. Overall, this perspective positions Notch signaling as a plausible, though not yet universally validated, component of exercise-associated adaptation and a priority target for future mechanistic investigation.

## 1 Introduction

The Notch signaling pathway is a highly conserved intercellular communication system that regulates cell differentiation, proliferation, stem-cell maintenance, and context-dependent metabolic homeostasis (Salazar et al., 2020). In the nervous system, Notch signaling plays established roles in neural stem-cell maintenance, neurodevelopment, and selected aspects of neurogenesis and synaptic plasticity (Salazar et al., 2020; Gozlan and Sprinzak, 2023; Lampada and Taylor, 2023). Outside the nervous system, it has also been implicated in glucose regulation, lipid metabolism, redox balance, and tissue remodeling, although these functions are highly context-dependent and are often inferred from developmental or disease-associated models (Adams and Jafar-Nejad, 2019; Xu and Wang, 2021; Angelopoulos et al., 2022). Accordingly, Notch signaling is biologically relevant to both neural and metabolic homeostasis; however, the mechanisms linking exercise stimuli to tissue-specific Notch responses remain incompletely understood (Salazar et al., 2020; Gozlan and Sprinzak, 2023; Lampada and Taylor, 2023).

Substantial evidence indicates that exercise promotes neuroplasticity, enhances metabolic resilience, and may reduce susceptibility to neurodegenerative processes (Mahalakshmi et al., 2020; Bonanni et al., 2022). Exercise can also increase the expression of neurotrophic factors, including brain-derived neurotrophic factor (BDNF), nerve growth factor (NGF), and glial cell line-derived neurotrophic factor (GDNF), which are essential for neuronal survival, neuroplasticity, and neurogenesis (Mahalakshmi et al., 2020; Bonanni et al., 2022). However, these benefits are mediated by multiple interacting pathways, and the specific contribution of Notch signaling remains insufficiently defined (Mahalakshmi et al., 2020). Recent studies suggest that Notch signaling may participate in selected exercise-associated neural and metabolic adaptations, but current evidence does not support its classification as a universally established master regulator (Bonanni et al., 2022). In this review, terms such as “moderate” and “high-intensity” exercise are treated as individualized physiological domains rather than fixed workload categories and should therefore be interpreted relative to markers such as lactate thresholds, ventilatory thresholds, heart-rate reserve, oxygen uptake reserve, and perceived exertion, because baseline fitness substantially influences internal load (MacIntosh et al., 2021; Warner et al., 2022). Notch signaling appears to be involved in neural stem-cell activation and the regulation of neuroplasticity, and exercise intensity may exert bidirectional effects on Notch activity (Imayoshi et al., 2010). For example, strenuous exercise may increase oxidative and inflammatory stress, whereas moderate exercise often favors adaptive responses; however, whether these physiological differences translate into consistent, tissue-specific patterns of Notch activation remains unclear (Mahalakshmi et al., 2020; Bonanni et al., 2022; Pahlavani, 2022); Collectively, these observations suggest that the role of Notch signaling in exercise adaptation may be context-dependent, thereby providing a useful framework for understanding how exercise influences neural and metabolic health.

Accordingly, a critical evaluation of Notch-related mechanisms in exercise-associated neural and metabolic adaptation may help distinguish direct evidence from extrapolation and identify areas in which mechanistic support remains limited. This review examines the potential roles of Notch signaling in exercise-related neural and metabolic regulation from molecular to systems-level perspectives, while emphasizing evidential strength, tissue specificity, and mechanistic gaps. By synthesizing the diverse roles of Notch signaling in neural and metabolic biology, we aim to provide a hypothesis-generating framework for future mechanistic research rather than a definitive rationale for immediate Notch-targeted therapeutic intervention.

## 2 Molecular mechanisms, and multifunctionality of Notch signaling pathway

### 2.1 Activation mechanisms of Notch signaling

**Figure 1:**
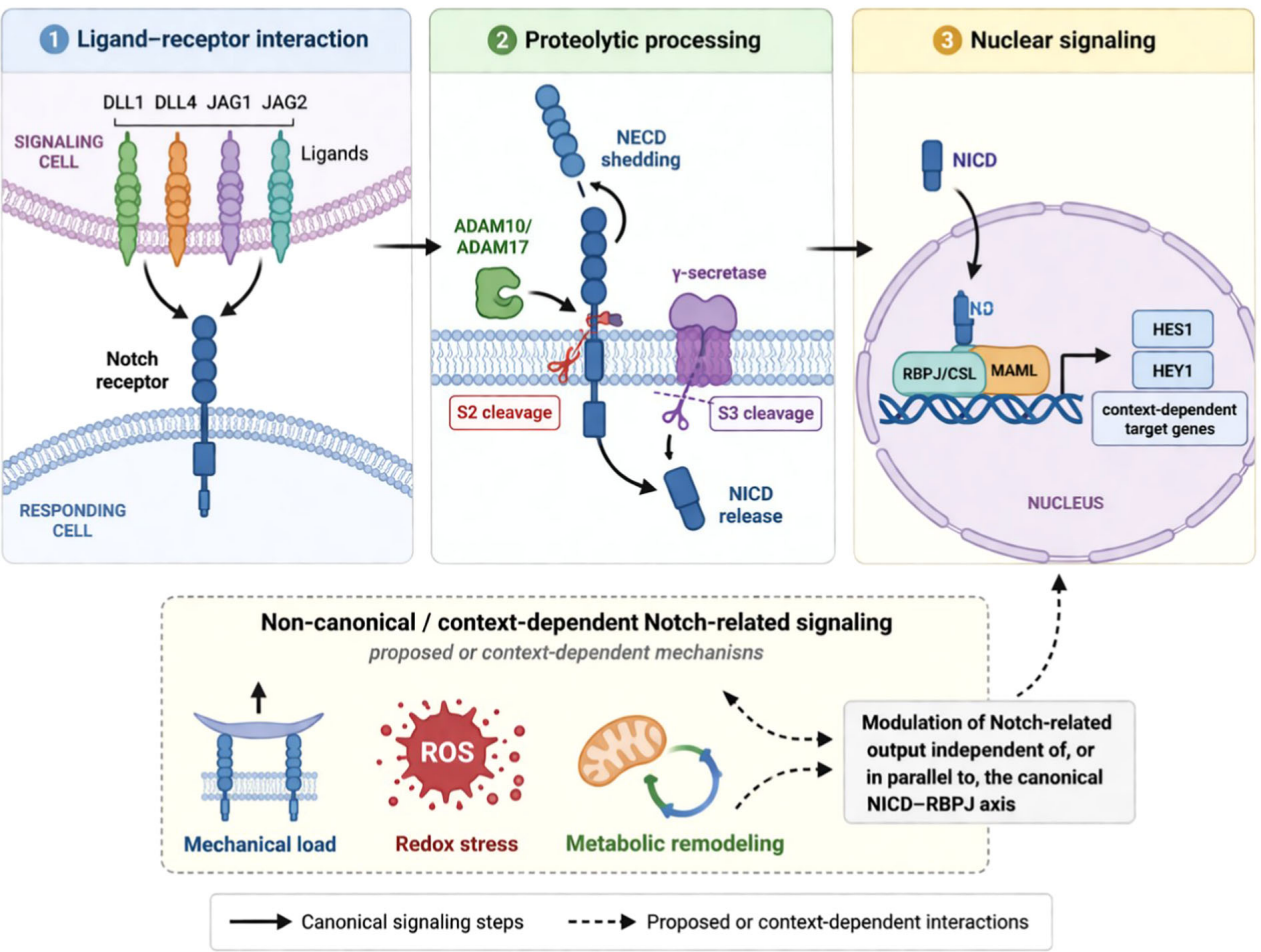
Canonical activation sequence and context-dependent extensions of Notch signaling. This figure depicts the canonical activation sequence of Notch signaling together with selected context-dependent extensions. In the canonical pathway, membrane-bound ligands, such as DLL1, DLL4, JAG1, and JAG2, expressed on signaling cells bind to Notch receptors on neighboring cells and thereby initiate receptor activation. This process is followed by sequential proteolytic cleavages, including S2 cleavage mediated by ADAM family metalloproteases, such as ADAM10 and ADAM17, and S3 cleavage mediated by the γ-secretase complex, ultimately resulting in the release of the Notch intracellular domain (NICD). NICD subsequently translocates to the nucleus, where it interacts with RBPJ/CSL and co-activators such as MAML to regulate the transcription of target genes, including members of the HES and HEY families. In addition to this canonical cascade, Notch-related signaling may be modulated by context-dependent mechanisms, including mechanical cues, redox status, and metabolic conditions. These influences may alter Notch signaling output either in parallel with or independently of the classical NICD–RBPJ axis. Such non-canonical or context-dependent interactions are not yet uniformly defined and may vary across cell types and physiological states. Overall, the figure highlights both the well-established core signaling sequence and the broader regulatory context in which Notch pathway activity may be modulated.

The Notch signaling pathway is a conserved intercellular communication mechanism that transmits signals through direct receptor–ligand contact. The Notch receptor, a transmembrane protein, undergoes a distinctive multistep activation process involving ligand binding, receptor cleavage, and the release of signaling fragments (**Figure 1**).

#### 2.1.1 Receptor-ligand interaction

In the Notch signaling pathway, ligands expressed on neighboring cells, such as Delta and Jagged, bind directly to Notch receptors on target cells, thereby initiating the signaling cascade (Hori et al., 2013). Ligand binding induces a conformational change in the Notch receptor, which exposes the S2 cleavage site to proteolysis and ultimately leads to the release of the Notch intracellular domain (NICD) (Hori et al., 2013). NICD subsequently translocates to the nucleus, where it regulates the transcription of downstream target genes (Hori et al., 2013). This signaling cascade is essential for a wide range of developmental and physiological processes and is mediated in part by interactions between the DSL domain of the ligand and the Notch receptor (Hori et al., 2013).

#### 2.1.2 Receptor cleavage, and release of signaling fragments

Following ligand binding, the Notch receptor undergoes a series of proteolytic cleavage events. Members of the ADAM family of metalloproteases, particularly ADAM10 and, in some contexts, ADAM17, mediate S2 cleavage at the plasma membrane, thereby generating a membrane-tethered intermediate that is subsequently processed by the γ-secretase complex (Kopan and Ilagan, 2009; Henrique and Schweisguth, 2019). This intermediate is then cleaved at the S3 site, resulting in the release of the NICD (Henrique and Schweisguth, 2019; Garis and Garrett-Sinha, 2020). NICD subsequently translocates to the nucleus, where it interacts with RBPJ (also known as CSL) and co-activators such as MAML to initiate the transcriptional activation of target genes. This sequence illustrates the distinctive contact-dependent nature of Notch signaling and the effectively irreversible character of its signal transduction process (Henrique and Schweisguth, 2019; Garis and Garrett-Sinha, 2020).

#### 2.1.3 Downstream signaling molecules HES1, and HEY1

Upon ligand-induced activation of the Notch receptor, NICD is released by γ-secretase and translocates to the nucleus (Kopan and Ilagan, 2009). There, NICD forms a transcriptional activation complex with the DNA-binding protein RBPJ and co-activators such as MAML, thereby inducing the expression of downstream target genes, including the key transcriptional repressors HES1 and HEY1, which play important roles in regulating cell proliferation, differentiation, and fate determination (Henrique and Schweisguth, 2019; Friedrich et al., 2022). Importantly, signaling output is further shaped by receptor–ligand composition, as DLL1, DLL4, JAG1, and JAG2 can produce distinct signaling dynamics and biological effects across tissues (Hori et al., 2013; Gozlan and Sprinzak, 2023). In neural stem cells (NSCs), HES1 is essential for maintaining the undifferentiated state and preventing premature differentiation by repressing neuronal differentiation genes, thereby preserving stem-cell pool stability (Kageyama et al., 2007; Riya et al., 2022). In transgenic mouse models overexpressing HES1, elevated HES1 levels promote neural stem-cell expansion and prolong neurogenesis, thereby stabilizing the postembryonic neural stem-cell pool (Ohtsuka and Kageyama, 2021). Similarly, HEY1 inhibits specific differentiation pathways in multiple cell types and is important for organ development and tissue regeneration (Li et al., 2012). In addition to the canonical NICD–RBPJ–MAML axis, non-canonical and RBPJ-independent Notch-related signaling has also been described. These alternative mechanisms may be relevant in exercise-related contexts involving mechanical load, oxidative stress, or metabolic remodeling (Stassen et al., 2020; Friedrich et al., 2022; Zhou et al., 2022).

### 2.2 Roles of Notch in neurons, and glial cell

The Notch signaling pathway plays multifaceted roles in neural development, particularly in the generation, differentiation, and proliferation of neurons and glial cells.

During early neural development, Notch signaling prevents the premature differentiation of neural stem cells (NSCs) by regulating downstream transcription factors such as HES1 and HEY1, thereby preserving their undifferentiated state (Imayoshi et al., 2010; Shimojo et al., 2011). Upon ligand–receptor interaction, such as Delta–Notch or Jagged–Notch binding, the NICD is released and translocates to the nucleus, where it associates with co-activators to induce the expression of HES and HEY genes (Fischer and Gessler, 2007). As transcriptional repressors, HES1 and HEY1 suppress proneural genes, including *Neurogenin2*, thereby maintaining NSCs in an undifferentiated state (Imayoshi et al., 2010; Shimojo et al., 2011).

The dynamic regulation of Notch signaling is also critical for NSC fate determination during cell division. NSCs can divide asymmetrically and transmit distinct Notch activity states to their progeny, thereby influencing whether daughter cells adopt neuronal or glial fates (Lampada and Taylor, 2023).For example, when Notch signaling remains active in one daughter cell but is reduced in the other, the Notch-active cell tends to retain stem-cell properties, whereas the other is more likely to differentiate into a neuron. This asymmetry helps preserve the NSC pool and maintain tissue homeostasis (Imayoshi et al., 2010; Lampada and Taylor, 2023). Moreover, appropriate Notch activity is essential for balanced neurogenesis during development. Both excessive and insufficient Notch activation can disrupt the balance between neuronal and glial differentiation, thereby impairing normal neural development and function (Shimojo et al., 2011; Lampada and Taylor, 2023). Overall, Notch signaling is a key regulator of NSC maintenance and differentiation, ensuring the cellular and structural stability required for orderly neural development. In addition to its role in NSCs, Notch signaling also contributes to the differentiation of oligodendrocyte progenitor cells (OPCs) and astrocytes, thereby helping to maintain functional balance within the central nervous system (Lampada and Taylor, 2023; Tran et al., 2023). Specifically, Notch signaling regulates OPC proliferation and their transition into mature oligodendrocytes, thereby influencing myelination (Brosnan and John, 2009).

### 2.3 Notch, and metabolic influence

Notch signaling plays important roles not only in cell differentiation and development but also in the regulation of cellular metabolism. Its metabolic functions have attracted increasing attention, particularly in relation to glucose metabolism, lipid metabolism, and oxidative stress homeostasis.

#### 2.3.1 Glucose metabolism

Accumulating evidence indicates that the Notch signaling pathway contributes to metabolic regulation in NSCs and cancer stem cells, including GSCs. In these cells, Notch signaling appears to support adaptation to the local microenvironment by helping meet metabolic demands, particularly through effects on glycolysis and mitochondrial function (Tang et al., 2021). In rapidly proliferating NSCs and GSCs, Notch signaling can promote glucose uptake by regulating the expression of key transport proteins such as GLUT1, thereby supporting the high energy demands of metabolically challenging environments, including the tumor microenvironment (Teodorczyk and Schmidt, 2014; Contreras et al., 2018).

In addition, Notch signaling regulates the balance between cell survival and differentiation under varying metabolic conditions. In NSCs and GSCs, Notch activity helps maintain stem-cell characteristics and may enhance cellular adaptation and survival in specific microenvironments. This effect is particularly evident in tumor settings, where Notch signaling has been associated with suppression of apoptosis, maintenance of self-renewal, and inhibition of differentiation, thereby supporting the continued growth and proliferation of cancer stem cells (Teodorczyk and Schmidt, 2014; Contreras et al., 2018). Recent studies have further explored the crosstalk between Notch signaling and other metabolic pathways, including the glucose dependency observed in tumor cells. As understanding of the metabolic functions of Notch signaling deepens, targeted therapies directed at this pathway, including γ-secretase inhibitors, have emerged as potential therapeutic strategies (Teodorczyk and Schmidt, 2014).

#### 2.3.2 Lipid metabolism influence

The role of Notch signaling in lipid metabolism has also received increasing attention, particularly with respect to lipid synthesis and degradation. Available evidence suggests that Notch signaling contributes to membrane stability and the dynamic regulation of fatty-acid metabolism and storage by modulating the expression of key metabolic enzymes and transcription factors. In particular, Notch signaling is closely associated with lipogenic transcription factors such as SREBP1c, which promote lipid synthesis in the liver and other tissues and regulate lipid accumulation in hepatocytes (Adams and Jafar-Nejad, 2019; Xu and Wang, 2021). Moreover, through its interaction with the mTOR pathway, Notch signaling may influence cellular nutrient responses and thereby regulate lipid synthesis and breakdown. In models of non-alcoholic fatty liver disease (NAFLD), Notch activation has been shown to promote lipid accumulation and aggravate hepatic lipid metabolic disorders, largely through mTORC1-related mechanisms (Xu and Wang, 2021). Regulation of lipid metabolism is particularly important for the long-term survival of neurons, as stable lipid homeostasis helps preserve membrane integrity and cellular function. In this context, Notch signaling may also contribute to energy balance by ensuring an adequate lipid supply during stress or metabolic challenge, thereby supporting long-term neuronal survival and normal function (Adams and Jafar-Nejad, 2019).

#### 2.3.3 Oxidative stress balance

Notch signaling also plays a role in cellular antioxidant responses, particularly in resistance to oxidative stress induced by reactive oxygen species (ROS). Previous studies have suggested that Notch signaling may reduce ROS production by regulating the expression of antioxidant genes, thereby limiting oxidative damage in neural cells and counteracting neurodegenerative changes associated with oxidative stress (Hannan et al., 2020; Franzoni et al., 2021). Evidence also indicates crosstalk between the Notch and Nrf2 pathways, through which Notch signaling may promote the expression of antioxidant enzymes such as heme oxygenase-1 (HO-1) and glutathione-related enzymes, thereby enhancing the antioxidant capacity of neural cells and suppressing neuroinflammation (Hannan et al., 2020). In addition, Notch signaling may counteract ROS accumulation and ROS-induced injury by modulating pathways in neurons and microglia, including PI3K/Akt signaling. This mechanism may be particularly relevant in neurodegenerative disorders such as Parkinson’s disease and Alzheimer’s disease (AD) (Hannan et al., 2020; Jembrek, 2024). From this perspective, modulation of Notch signaling may represent a potential neuroprotective strategy by attenuating ROS-driven oxidative damage and reducing neural cell apoptosis through limitation of neuroinflammatory responses (Hannan et al., 2020; Jembrek, 2024). Collectively, these findings underscore the potential protective role of Notch signaling in neurodegenerative disease and highlight its relevance to research on antioxidant-based interventions.

## 3 Activation, and regulatory mechanisms of Notch signaling in exercise

### 3.1 The impact of exercise on Notch signaling

The relationship between exercise and Notch signaling has attracted increasing attention; however, the available evidence should be interpreted with caution because direct causal data remain limited (Bonanni et al., 2022; Lampada and Taylor, 2023). Several studies have reported exercise- or exercise-like stimulus-associated changes in Notch-related readouts, particularly in neural stem-cell and regenerative contexts; however, these observations do not establish a universal exercise-to-Notch activation pathway (Bonanni et al., 2022; Lampada and Taylor, 2023).

#### 3.1.1 Activation of Notch signaling by exercise

Physical activity, particularly aerobic exercise, is associated with enhanced hippocampal neurogenesis and neuroplasticity, and some of these adaptations may intersect with Notch-related signaling (Liu and Nusslock, 2018; Bonanni et al., 2022; Lampada and Taylor, 2023). Current evidence suggests that aerobic exercise may influence NOTCH1- and HES1-related pathways in specific contexts; however, the available data do not support the conclusion that exercise-induced neuronal proliferation and cognitive improvement are primarily mediated by Notch activation alone (Liu and Nusslock, 2018; Schoenfeld and Swanson, 2021; Lampada and Taylor, 2023). Exercise may promote the proliferation and survival of hippocampal neural stem cells in the dentate gyrus, but whether these effects require direct Notch1 activation remains unresolved (Imayoshi et al., 2010). More plausibly, exercise-related neurogenesis and functional recovery may involve coordinated interactions between Notch-related pathways and other exercise-responsive factors, including angiogenic and neurotrophic mediators such as BDNF and IGF-1, which collectively support neurogenesis and brain plasticity (Chieffi et al., 2017; Liu and Nusslock, 2018). Some studies further suggest that exercise-associated Notch-related signaling may contribute to the attenuation of age-related cognitive decline in older animals. In addition, the neurogenic effects of exercise are closely linked to antidepressant-like effects and emotional stability, indicating that aerobic exercise may benefit not only cognition but also emotional and mental health (Chieffi et al., 2017).

Overall, Notch signaling may represent one component of the regulatory network underlying exercise-associated neurogenesis in the central nervous system, but current evidence does not justify defining it as a singular or universally dominant mechanism. Rather, this pathway should be viewed as a plausible contributor that warrants further mechanistic investigation in the context of exercise-based interventions for neurodegenerative disorders.

#### 3.1.2 Influence of neural stem cell Notch signaling by exercise

During exercise-associated NSC proliferation, Notch signaling may exert an important regulatory role. Notch signaling helps maintain the undifferentiated state of NSCs and preserve their proliferative capacity by modulating multiple receptors and downstream effectors, including HES5 (Shimojo et al., 2011).For example, aerobic exercise has been reported to increase NSC activity in mouse models while preserving stem-cell identity through Notch1-related signaling, thereby promoting self-renewal, preventing premature differentiation, extending neurogenesis, and supporting neuroregeneration (Sood et al., 2022; Lampada and Taylor, 2023).

Notch signaling in NSCs appears to shift dynamically between quiescent and active states, thereby influencing neurogenesis. In particular, Notch1 has been associated with the maintenance of active NSCs, whereas Notch2 is thought to regulate the transition to quiescence and thereby prevent premature depletion of the NSC pool. Additional evidence suggests that the coordinated effects of Notch1 and Notch2 may involve downstream transcription factors such as HES5, whose expression may increase after aerobic exercise and may contribute to enhanced NSC proliferation (Sood et al., 2022).

Taken together, these findings suggest that exercise-associated Notch signaling may support NSC renewal and proliferation and may thereby contribute to neuroregeneration, with potential relevance to brain development and injury repair.

#### 3.1.3 Bidirectional regulation impacts of exercise on Notch signaling

Aerobic exercise appears to exert complex, context-dependent effects on Notch signaling, and moderate- and high-intensity exercise may influence this pathway differently. Moderate aerobic exercise is often associated with adaptive physiological responses, but whether these responses involve direct activation of Notch signaling depends on the tissue, cell type, and molecular readout under investigation (Pahlavani, 2022; Lampada and Taylor, 2023; Zhang et al., 2023). For example, some studies indicate that aerobic exercise may coincide with Notch1-related signaling in neural or cardiac adaptive contexts; however, such observations do not establish a universal causal role for Notch1 in reducing disease risk (Pahlavani, 2022).

By contrast, strenuous or prolonged exercise may increase oxidative, inflammatory, and mechanical stress in ways that alter the biological significance of Notch-related responses (Pahlavani, 2022; Luo et al., 2024). Some reports suggest that high-intensity exercise may be associated with reduced Notch1-related readouts in specific experimental settings, potentially in parallel with increased oxidative or inflammatory stress. In certain models, altered Notch-related signaling has been associated with maladaptive cardiac remodeling, including fibrosis; however, these findings remain tissue- and model-dependent (Bo et al., 2020; Zhang et al., 2023). Such signaling changes may coincide with increased apoptosis or inflammatory cascades in some contexts, but the causal sequence remains incompletely defined.

**Figure 2:**
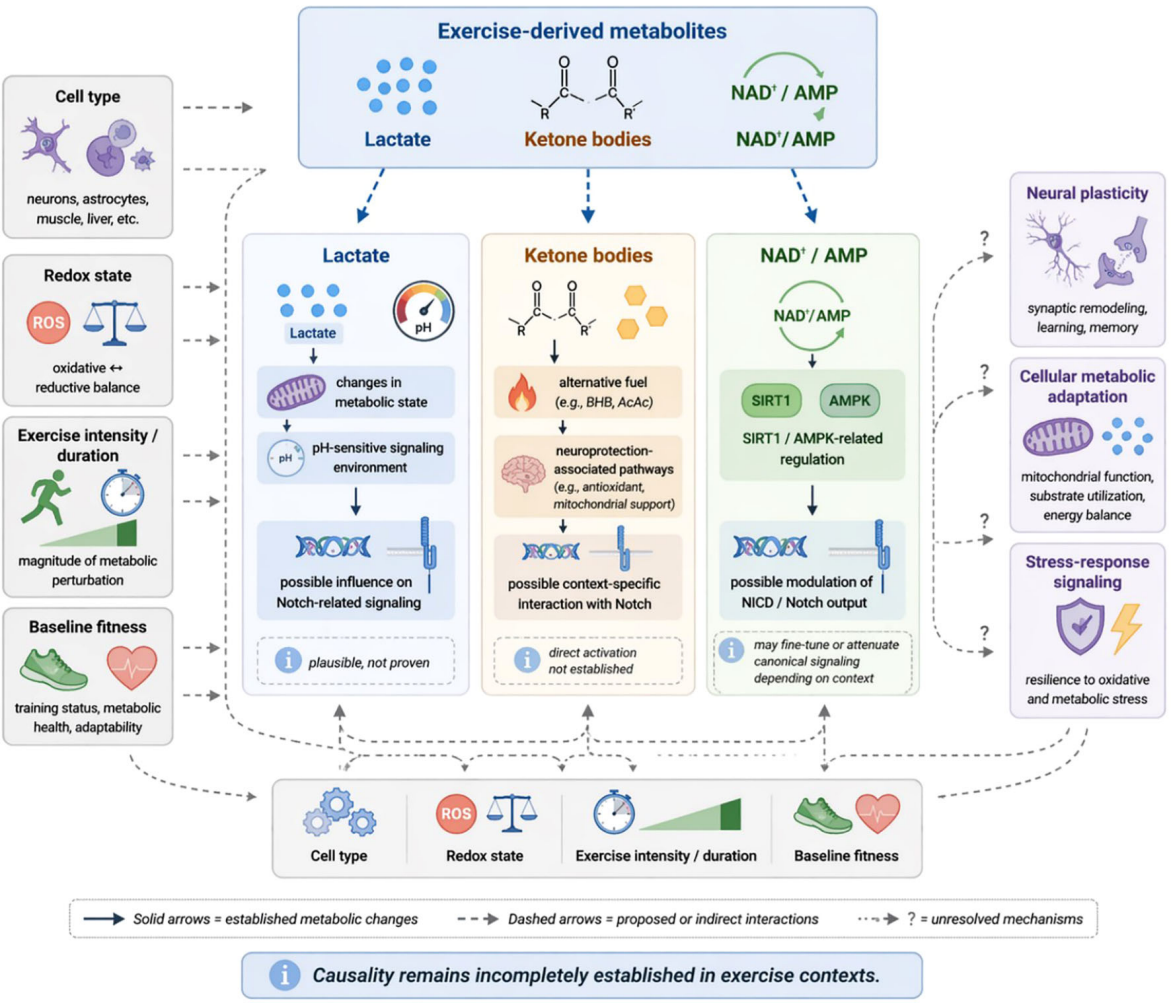
Context-dependent interactions between exercise stimuli and Notch signaling across tissues. This figure presents a context-dependent framework in which exercise stimuli, including intensity, duration, and modality, may interact with Notch signaling across multiple tissues, including the brain, skeletal muscle, and heart. In neural stem cells, exercise-associated changes may coincide with alterations in Notch-related signaling and neurogenesis; however, direct causal relationships remain insufficiently established. In skeletal muscle, Notch signaling is more consistently associated with satellite-cell activation and muscle regeneration, although these responses are time-dependent and context-specific. In cardiac tissue, Notch-related responses have been reported in models of exercise-induced remodeling, but the findings remain variable and model-dependent. These interactions may be further modulated by factors such as redox status, exercise-derived metabolites, including lactate and ketone bodies, and baseline fitness. Overall, the figure emphasizes that exercise–Notch relationships are not uniform, but instead depend on tissue type, cellular context, and physiological conditions, with several underlying mechanisms remaining uncertain.

Overall, the effects of exercise intensity on Notch-related signaling are best interpreted within a context-dependent framework in which tissue type, cell type, receptor-ligand composition, oxidative burden, and baseline fitness shape the response, rather than as a universal bidirectional rule (Pahlavani, 2022; Gozlan and Sprinzak, 2023; Lampada and Taylor, 2023).These observations underscore the need for future studies to determine whether Notch signaling is causal, compensatory, or merely correlative in exercise-related adaptation (**Figure 2**).

### 3.2 Exercise modality, intensity, and the context-dependent nature of Notch-related responses

Different exercise modalities and internal-load domains may be associated with distinct Notch-related responses; however, current evidence does not support a uniform pattern of exercise–Notch activation across tissues. **Table 1** summarizes representative evidence linking exercise or exercise-relevant contexts to Notch-related signaling in different tissues, while distinguishing direct exercise-associated observations from mechanistic evidence derived from non-exercise models.

**Table 1:** Representative evidence linking exercise or exercise-relevant contexts to Notch-related signaling.

Study/reference	Model	Exercise modality or stimulus	Intensity/load description	Tissue/cell type	Notch-related readout	Main finding	Key limitation/evidence level
Revelo Herrera and Leon-Rojas, 2024; Pickersgill et al., 2022; Mang et al., 2013	Human and animal studies summarized in reviews	Aerobic exercise/endurance-type training	Often variably defined across studies; not consistently reported using unified internal-load metrics	Brain, especially hippocampal-related contexts	Indirect Notch-related interpretation; often neuroplasticity outcomes rather than direct pathway measurement	Aerobic exercise is consistently associated with improved neuroplasticity and cognition; some findings are compatible with possible involvement of Notch-related signaling	Mostly indirect evidence; many studies do not directly measure Notch components or manipulate the pathway
Liu and Nusslock, 2018; Bonanni et al., 2022; Lampada and Taylor, 2023	Review-level synthesis of rodent and mechanistic literature	Exercise-associated neurogenesis context	Relative exercise intensity often not resolved for Notch-specific interpretation	Hippocampus/neural stem-cell niche	NOTCH1-, HES1-, neurogenesis-related interpretation	Exercise may intersect with Notch-related pathways in neural stem-cell regulation and neurogenesis	Largely inferential; Notch is one candidate pathway among many
Imayoshi et al., 2010	Mouse genetic study	Not exercise-specific; adult and developing brain stem-cell biology	Not applicable	Neural stem cells	Notch pathway requirement in NSC maintenance	Demonstrates that Notch is essential for NSC maintenance, supporting biological plausibility for exercise-related relevance	Strong mechanistic Notch evidence, but not direct exercise evidence
MacKenzie et al., 2013	Human skeletal muscle after acute resistance exercise	Acute resistance exercise	High-load acute bout; protocol-specific	Skeletal muscle	Notch activation/myostatin-related signaling	Acute resistance exercise is associated with transient Notch-related responses in skeletal muscle	Stronger exercise-linked evidence than in CNS, but limited to acute muscle context
Bjornson et al., 2012; Mourikis et al., 2012	Adult muscle stem-cell models	Regeneration/stem-cell quiescence context, not direct exercise intervention	Not applicable	Satellite cells/skeletal muscle stem cells	Canonical Notch signaling in quiescence maintenance	Supports a clear role for Notch in satellite-cell quiescence and self-renewal	Strong mechanistic support for muscle stem-cell biology, but not direct exercise protocol evidence
Pahlavani, 2022	Review of cardiac exercise signaling pathways	Aerobic exercise/exercise-associated cardioprotection	Variable across included studies	Cardiac tissue/cardiomyocytes	Notch1-associated cardioprotective signaling	Some studies suggest Notch-related pathways may be associated with adaptive cardiac remodeling	Mostly review-level and model-dependent; direct causality remains limited
Zhang et al., 2023	Mouse voluntary running model	Voluntary running	Physiological loading; not directly comparable to fixed “moderate/high-intensity” human categories	Heart	Notch1/p38 signaling	Notch1-associated signaling was implicated in physiological cardiac hypertrophy after voluntary running	Organ-specific finding; does not establish a universal exercise–Notch rule
Bo et al., 2020	Review/experimental cardiac regeneration contexts	Aerobic exercise-associated cardiac remodeling	Variable	Heart	Notch-related remodeling interpretation	Altered Notch-related signaling has been associated with adaptive or maladaptive remodeling depending on context	Cardiac-specific and model-dependent; limited relevance to neural conclusions
Luo et al., 2024	Specific experimental model	Exercise-induced muscle adaptation context	High-intensity or stress-associated context	Muscle/inflammatory-metabolic context	Notch3-associated readouts	High-load conditions may be associated with altered Notch3-related inflammatory responses in specific models	Highly context-specific; should not be generalized as a universal intense-exercise Notch response
Wu et al., 2023; El Hayek et al., 2019	Brain metabolism/exercise-metabolite context	Exercise-associated lactate signaling	Often linked to higher metabolic load rather than standardized Notch-specific exercise intensity	Brain/neurons	Indirect Notch-related interpretation; lactate, metabolism, plasticity	Lactate is biologically relevant to brain metabolism and plasticity and may shape signaling contexts relevant to Notch	No direct proof that exercise-derived lactate activates neuronal Notch signaling
García-Rodríguez and Giménez-Cassina, 2021; Gough et al., 2021	Ketone-body and neuroprotection literature	Fasting/prolonged exercise/ketogenic context	Metabolic state rather than direct exercise-defined Notch intensity	Brain	Putative Notch-related interaction	Ketone bodies may support neuroprotection and metabolic adaptation, with possible context-specific interaction with Notch	Direct exercise–ketone–Notch evidence remains sparse
Guarani et al., 2011; Bai et al., 2018; Ji and Yeo, 2022	Endothelial, macrophage, and metabolic regulation contexts	NAD+/AMP/SIRT1/AMPK-associated signaling	Not exercise-specific in most cases	Multiple cell types	NICD acetylation/SIRT1-related modulation	NAD+/SIRT1-related mechanisms may modulate Notch output rather than simply activate it	Mechanistically informative, but mostly extrapolated to exercise contexts
Adams and Jafar-Nejad, 2019; Xu and Wang, 2021	Liver development/NAFLD-related models	Disease and metabolic remodeling contexts	Not exercise-specific	Liver	Notch in gluconeogenesis/lipid metabolism	Notch signaling is implicated in hepatic metabolic regulation, supporting plausibility for exercise relevance	Strong liver biology evidence, but limited direct exercise evidence
Angelopoulos et al., 2022; Chiappara et al., 2024	Neural stem-cell metabolism/oxidative stress contexts	Redox and metabolic stress contexts	Not exercise-specific	Neural cells/stem cells	Redox-associated Notch interpretation	Notch may interact with antioxidant and metabolic pathways	Mostly non-exercise evidence; causal relevance to exercise adaptation remains unresolved

#### 3.2.1 Aerobic exercise and putative Notch-related adaptation

Aerobic exercise, including jogging and swimming, is associated with beneficial effects on neuroplasticity and brain health, and some of these adaptations may intersect with Notch-related signaling. Several studies have reported increased hippocampal Notch-related markers following sustained aerobic training; however, the causal contribution of Notch signaling to these adaptations remains incompletely understood. For example, running-based interventions have been associated with altered hippocampal plasticity and, in some reports, with changes in Notch-related readouts. Nevertheless, these findings do not demonstrate that Notch1 activation is the primary driver of long-term potentiation (LTP) or broader neuroplasticity-related outcomes (Pickersgill et al., 2022; Revelo Herrera and Leon-Rojas, 2024).

Although Notch signaling is biologically relevant to neuroregeneration and plasticity, its specific contribution to exercise-associated adaptation should be interpreted cautiously. Exercise promotes neuronal connectivity and structural remodeling in the brain, thereby supporting cognition and memory through multiple interacting pathways. These adaptations may involve changes in neural stem-cell division, differentiation, and niche dynamics, but the extent to which they depend directly on canonical Notch signaling remains uncertain. Multiple studies suggest that regular aerobic exercise may interact with Notch-related pathways alongside other mediators, such as BDNF, to support hippocampal structure and function; however, direct exercise-induced activation of Notch has not been consistently demonstrated across models (Mang et al., 2013).

#### 3.2.2 Resistance exercise and skeletal-muscle Notch biology

Resistance exercise is more convincingly linked to Notch biology in skeletal muscle than in the central nervous system, particularly through the regulation of satellite-cell quiescence, activation timing, self-renewal, and regeneration (MacKenzie et al., 2013). Acute resistance exercise has been associated with transient changes in Notch-related signaling in muscle, likely reflecting regenerative and remodeling demands. However, these responses are time-sensitive and should not be interpreted as evidence of a generalized systemic pattern of Notch activation. Such transient responses may support muscle repair and stem-cell maintenance, and the mechanistic evidence for this role is currently stronger in skeletal muscle than in neural tissue (Bjornson et al., 2012; Mourikis et al., 2012; MacKenzie et al., 2013).

#### 3.2.3 Dose, duration, and temporal dependence

Exercise-associated Notch-related responses may vary according to exercise dose, frequency, modality, and sampling time. Acute or lower-load exercise may be associated with localized and short-lived signaling changes, whereas repeated training or prolonged loading may induce more persistent tissue responses through regeneration, metabolic adaptation, vascular remodeling, or altered niche dynamics. Some studies suggest that repeated aerobic loading may intersect with calcium-sensitive and stress-responsive pathways relevant to Notch biology in cardiovascular tissue, but the causal role of Notch in these adaptations remains incompletely defined (Pahlavani, 2022; Gozlan and Sprinzak, 2023).

In summary, exercise dose and frequency are likely important modifiers of Notch-related responses, but current data are insufficient to support prescriptive claims regarding how specific exercise loads consistently activate Notch signaling across tissues.

#### 3.2.4 Context dependence and the limits of a simple dose–response model

Different exercise intensities may be associated with distinct Notch-related outcomes, but these responses should be interpreted in relation to tissue type, cell state, receptor-ligand composition, redox burden, and baseline fitness rather than within a simple universal dose-response framework. Moderate exercise is often associated with adaptive cellular responses that support metabolic resilience, survival signaling, and tissue remodeling. In some contexts, these responses may involve Notch-related mechanisms, but direct causality has not been established consistently across tissues. Evidence suggests that moderate-intensity exercise may coincide with cardioprotective signaling patterns involving Notch1-associated pathways; however, these findings remain tissue- and model-specific and should not be generalized across all exercise contexts (Pahlavani, 2022). Conversely, strenuous or prolonged exercise may increase oxidative, inflammatory, and mechanical stress in ways that alter the biological significance of Notch-related readouts. Some reports have linked high-intensity or prolonged loading to altered Notch3-associated signaling and inflammatory responses in specific models, but these findings should not be interpreted as evidence of a universal maladaptive Notch program induced by intense exercise (Luo et al., 2024).

Thus, exercise intensity is best viewed as one modifier of Notch-related biology within a broader context-dependent framework, rather than as the basis for a simplified model in which moderate exercise uniformly activates beneficial Notch signaling and intense exercise uniformly suppresses it.

## 4 Role of Notch signaling pathway in exercise-associated metabolic influence

### 4.1 Adaptive influence of metabolism

**Figure 3:**
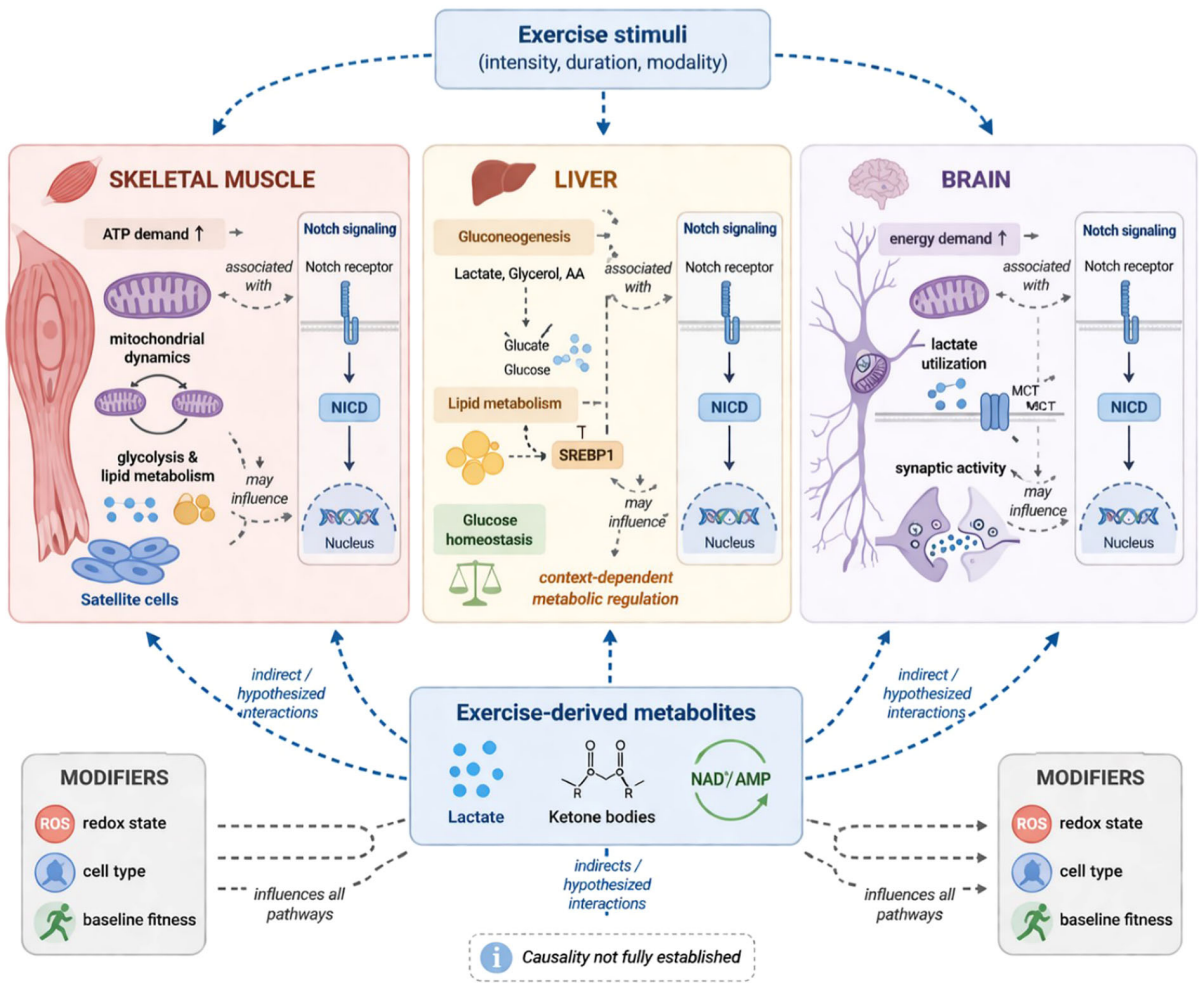
This figure summarizes the potential roles of Notch signaling in exercise-associated metabolic adaptation across skeletal muscle, liver, and brain within a context-dependent framework. In skeletal muscle, exercise increases ATP demand and is associated with changes in mitochondrial dynamics, glycolysis, and lipid metabolism; these processes may intersect with Notch-related signaling and satellite-cell regulation. In the liver, Notch signaling has been implicated in gluconeogenesis, lipid metabolism, and glucose homeostasis; however, its specific contribution to exercise-induced hepatic metabolic adaptation remains incompletely defined. In the brain, exercise-induced increases in energy demand and lactate utilization may coincide with signaling pathways relevant to synaptic activity and metabolic regulation, with possible, but as yet unconfirmed, involvement of Notch signaling. Exercise-derived metabolites, including lactate, ketone bodies, and NAD^+^/AMP-related pathways, may influence Notch-associated processes indirectly or in a context-specific manner, although direct causal relationships have yet to be established. These interactions are further shaped by modifiers such as redox status, cell type, and baseline physiological state. Overall, the figure emphasizes that Notch-related metabolic responses to exercise are tissue-specific and context-dependent and should often be regarded as hypothesis-generating rather than mechanistically confirmed.

During exercise, the Notch signaling pathway may contribute to metabolic adaptation across multiple organs, particularly skeletal muscle, liver, and brain (**Figure 3**).

#### 4.1.1 Energy metabolism in muscle

In response to exercise, skeletal muscle markedly increases ATP turnover to meet elevated energy demands, and Notch signaling may participate in this adaptive process. Previous studies suggest that Notch signaling supports ATP production by modulating mitochondrial function and related metabolic pathways, thereby helping to optimize energy supply (Xu et al., 2015). More specifically, Notch signaling has been linked to the regulation of mitochondrial dynamics and quality control, which are important for maintaining sustained energy production under conditions of high demand. Experimental evidence further indicates that Notch signaling may interact with mitochondria-associated endoplasmic reticulum membranes, potentially contributing to structural stability during ATP generation (Ito et al., 2023).

In addition, Notch signaling has been implicated in the regulation of glycolysis and lipid metabolism, thereby providing rapidly available energy substrates for muscle contraction and performance. Core components of the Notch pathway, such as DLL1, are highly expressed during muscle-cell differentiation and appear to contribute to skeletal-muscle regeneration and repair. Through these mechanisms, Notch signaling may help coordinate carbohydrate and lipid metabolism in response to the physiological demands of exercise (Zhang et al., 2021).

Overall, by modulating mitochondrial function and metabolic pathways, Notch signaling may support ATP production and facilitate glycolytic and lipid-metabolic adaptation during exercise. These effects may be relevant to both exercise performance and skeletal-muscle health.

#### 4.1.2 Influence of liver metabolism

In exercise-associated metabolic adaptation, Notch signaling may also play an important regulatory role in the liver. Existing evidence suggests that Notch signaling participates in the regulation of hepatic gluconeogenesis and lipid metabolism, particularly under the metabolic demands imposed by exercise. Some studies have reported that Notch signaling may help maintain blood-glucose homeostasis during exercise by promoting gluconeogenesis and thereby ensuring sufficient energy supply to skeletal muscle and other organs (Zhang et al., 2018a). In addition, Notch signaling has been linked to the regulation of lipid metabolism through effects on lipolysis and fatty-acid oxidation, processes that may contribute to sustained energy provision during prolonged exercise (Xu and Wang, 2021).

With respect to hepatic lipid metabolism, Notch signaling has been reported to regulate the expression of genes such as *SREBP1c*, thereby influencing the balance between lipogenesis and lipolysis. Experimental inhibition of Notch signaling has been associated with reduced hepatic lipid accumulation, suggesting potential therapeutic relevance for metabolic disorders such as non-alcoholic fatty liver disease (NAFLD) associated with obesity and diabetes (Valenti et al., 2013; Adams and Jafar-Nejad, 2019). These observations indicate that Notch-related metabolic regulation may be relevant both during and after exercise, potentially contributing to the restoration and maintenance of hepatic metabolic homeostasis (Adams and Jafar-Nejad, 2019; Xu and Wang, 2021). However, whether targeting the Notch pathway can reliably optimize exercise-related metabolic adaptation remains to be established.

#### 4.1.3 Influence of brain metabolism

The increase in cerebral energy demand during exercise may also intersect with Notch-related signaling. Some evidence suggests that, in neurons, Notch signaling may support ATP production through glycolytic pathways and may also contribute to energy homeostasis by influencing lactate metabolism in the brain (Wu et al., 2023).

During exercise, increased systemic and local metabolic demand is accompanied by elevated lactate availability. Lactate is not merely a by-product of muscle activity, but also serves as an important energy substrate and signaling molecule in the brain (El Hayek et al., 2019). Studies have shown that high-intensity interval training (HIIT) can increase lactate levels and may influence mitochondrial function in the brain, particularly processes related to ATP production and hippocampal synaptic plasticity. These effects have been linked to lactate-associated upregulation of *PGC-1α* and mitochondrial fusion proteins, which may improve mitochondrial quality and function under increased energetic demand (van Praag et al., 2014; Hu et al., 2021).

Furthermore, Notch signaling may participate in the regulation of brain energy metabolism under conditions of elevated neuronal activity. In energy-demanding regions such as the hippocampus, Notch-related pathways may interact with glycolytic and mitochondrial processes, potentially influencing mitochondrial fusion and fission dynamics in association with lactate metabolism (Lampada and Taylor, 2023; Wu et al., 2023). Such adaptive responses may be relevant to synaptic plasticity and, consequently, to learning and memory.

Overall, exercise supports brain energy demands through multiple complementary mechanisms, and Notch signaling may represent one component of this broader metabolic regulatory network that contributes to long-term neuronal function and resilience.

### 4.2 Metabolic balance, and neuroprotection

Exercise-induced metabolic stress can provoke oxidative stress, and Notch signaling may contribute to the maintenance of metabolic homeostasis and neuroprotection during this process.

#### 4.2.1 Influence of metabolic balance

The Notch signaling pathway is involved in metabolic regulation and antioxidant responses and appears to be important for maintaining physiological homeostasis during and after exercise. Previous studies suggest that Notch signaling regulates mitochondrial metabolism and the activity of antioxidant enzymes, including superoxide dismutase (SOD) and glutathione peroxidase (GPX), thereby helping to limit ROS production and reduce the risk of oxidative damage in neural cells (Angelopoulos et al., 2022; Chiappara et al., 2024).

In the absence of Notch signaling, antioxidant capacity may decline, leading to elevated ROS levels and metabolic imbalance, which may aggravate neuronal injury and increase the risk of neurodegenerative disease. Conversely, activation of Notch signaling may attenuate free-radical accumulation by supporting redox homeostasis, thereby promoting neuroprotection as well as neural stem-cell survival and neurogenesis (Angelopoulos et al., 2022; Chiappara et al., 2024). Together, these metabolic and antioxidant regulatory functions suggest that Notch signaling may play an important role in neural health and stress adaptation and may represent a potential therapeutic target in neurodegenerative disorders.

#### 4.2.2 Neuroprotective functions

Notch signaling may also contribute to neuroprotection by regulating not only oxidative stress responses but also apoptosis-related pathways. In models of neurodegenerative disease and neuronal injury, increased Notch signaling has been associated with enhanced neuronal survival, partly through suppression of oxidative stress-induced cell death. More specifically, Notch1 signaling has been reported to reduce oxidative neuronal injury by inhibiting the ASK1-MKK3/6-p38 MAPK pathway. Through this mechanism, Notch1 signaling may suppress oxidative stress-related inflammation and apoptosis, thereby reducing neuronal damage (Mo et al., 2013; Zhang et al., 2018b). In addition, some studies suggest that Notch signaling may participate in exercise-associated neuroprotection by contributing to the attenuation of neuroinflammation and oxidative damage, thereby facilitating neuronal recovery (Mo et al., 2013).

These observations provide a useful molecular framework for understanding the neural benefits of exercise, while also highlighting Notch signaling as a plausible pathway involved in exercise-associated neuroprotection.

### 4.3 Exercise-driven metabolites as activators of Notch signaling

**Figure 4:**
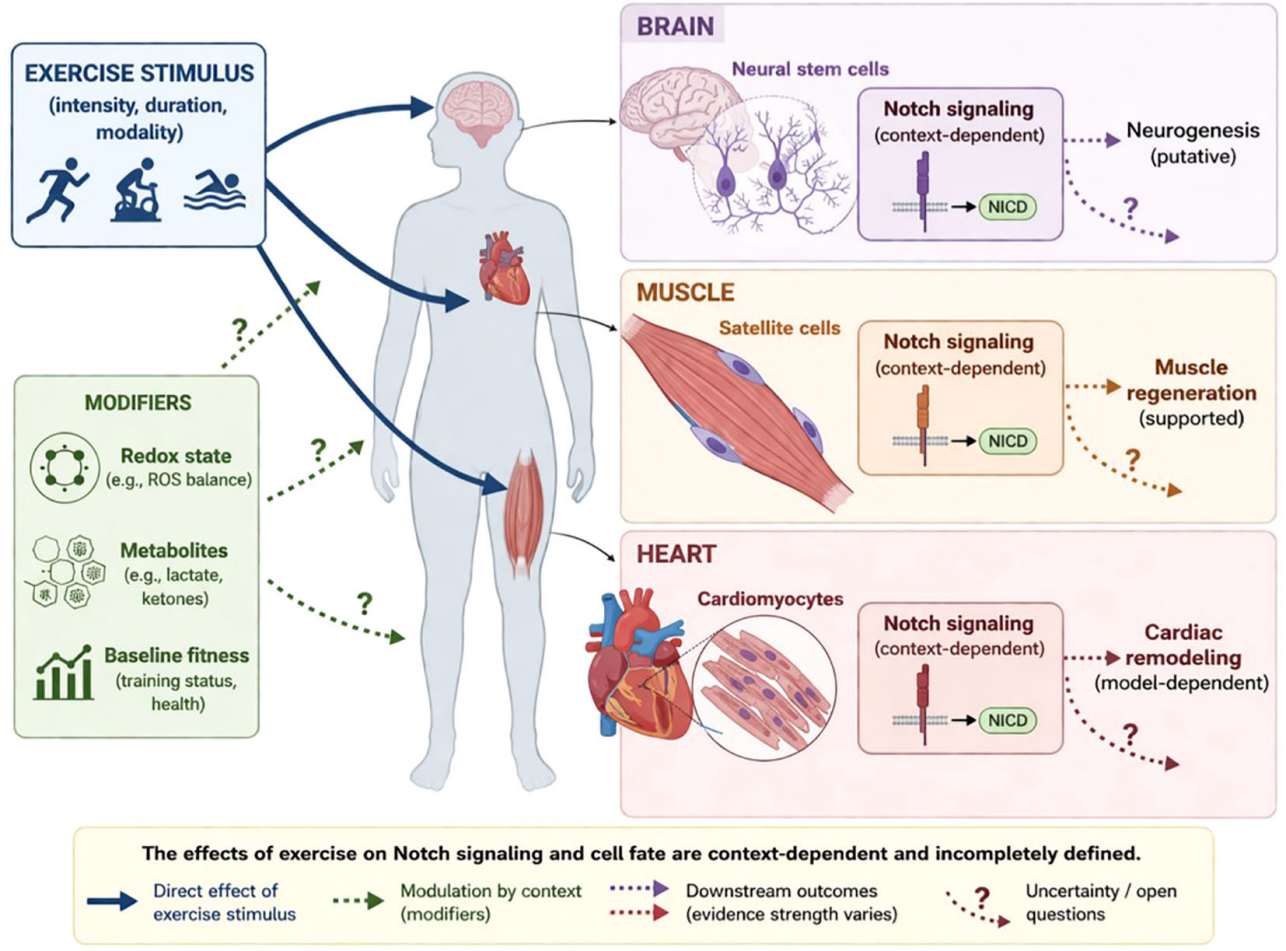
Putative and context-dependent interactions between exercise-derived metabolites and Notch-related signaling. This figure summarizes the proposed interactions between exercise-derived metabolites and Notch-related signaling within a context-dependent framework. Exercise increases the production of metabolites such as lactate, ketone bodies, and NAD^+^/AMP-related signals, all of which are known to influence cellular metabolic states and signaling environments. Lactate may alter intracellular pH and metabolic conditions, thereby shaping signaling contexts that could affect Notch-related pathways, although direct activation has not been demonstrated. Ketone bodies serve as alternative energy substrates and may engage pathways associated with neuroprotection; however, their interaction with Notch signaling remains speculative and context-dependent. NAD^+^/AMP-related pathways, through regulators such as SIRT1 and AMPK, may modulate Notch signaling dynamics rather than directly activate the pathway, potentially fine-tuning or attenuating signaling output depending on the cellular context. These interactions are further shaped by factors including cell type, redox status, exercise intensity and duration, and baseline physiological state. Accordingly, downstream outcomes such as neural plasticity, metabolic adaptation, and stress-response signaling should be regarded as potential indirect associations rather than confirmed Notch-dependent effects. Overall, the figure emphasizes that metabolite–Notch interactions in exercise settings remain incompletely understood and should be viewed as hypothesis-generating rather than mechanistically established.

Some metabolites generated during exercise may directly or indirectly modulate Notch signaling, thereby providing additional insight into the potential role of Notch in exercise-related adaptation (**Figure 4**).

#### 4.3.1 Lactate

As an important metabolite generated during exercise, lactate has emerged as a signaling molecule with functions that extend beyond its role in energy metabolism. Lactate not only serves as an energy substrate but may also influence cell metabolism and recovery-related signaling; however, direct evidence that exercise-derived lactate activates Notch signaling in neurons or glial cells remains limited (El Hayek et al., 2019; Wu et al., 2023). A more cautious interpretation is that lactate-associated shifts in cellular metabolism and pH may shape signaling environments in a context-dependent manner, whereas severe acidosis is generally pathological and may impair protein function. Accordingly, lactate–Notch crosstalk in exercise should currently be regarded as plausible but unproven (El Hayek et al., 2019; Wu et al., 2023; Romero-Moreno et al., 2024).

#### 4.3.2 Ketone bodies

During prolonged aerobic exercise or fasting, ketogenesis increases substantially. This process primarily involves the β-oxidation of fatty acids to generate acetyl-CoA, which is subsequently converted into ketone bodies such as β-hydroxybutyrate (BHB) and acetoacetate (AcAc). These ketone bodies serve as alternative energy substrates for skeletal muscle and the brain when glucose availability is low, particularly when glycogen stores in muscle and liver are depleted, such as during fasting or prolonged exercise (García-Rodríguez and Giménez-Cassina, 2021; Gough et al., 2021).

In the brain, ketone bodies function not only as metabolic fuels but may also contribute to neuroprotection; however, direct evidence that exercise-induced ketone bodies regulate brain adaptation through Notch signaling remains limited (García-Rodríguez and Giménez-Cassina, 2021). Although the role of Notch signaling in cell differentiation, survival, and metabolic adaptation is well established, current evidence does not support a definitive conclusion that exercise-induced ketone bodies directly activate Notch signaling to promote neuronal metabolic adaptation. A more cautious interpretation is that ketone bodies may influence cellular conditions relevant to Notch-associated processes, particularly under fasting or low-glucose states, but such interactions remain context-dependent and mechanistically unresolved (García-Rodríguez and Giménez-Cassina, 2021).

Furthermore, the neuroprotective effects of ketone bodies have been highlighted in multiple studies. Ketone bodies may reduce neuroinflammation, enhance antioxidant defenses, and protect neurons by modulating intracellular calcium homeostasis and attenuating oxidative stress-related injury. Through these mechanisms, ketone bodies may support post-exercise brain function, cognitive health, and recovery; however, their proposed role as direct activators of Notch signaling remains speculative (Gough et al., 2021).

#### 4.3.3 Other metabolites

During exercise, the levels of small metabolites such as NAD^+^ and AMP increase substantially, particularly in skeletal muscle. NAD^+^ may influence Notch-related signaling through sirtuin-dependent mechanisms, but this relationship is context-dependent and should not be reduced to a uniform activating pathway (North and Verdin, 2004; Guarani et al., 2011; Bai et al., 2018).Sirtuins require NAD^+^ for their deacetylase activity, through which they regulate cellular metabolic homeostasis (North and Verdin, 2004). For example, elevated NAD^+^ levels may support mitochondrial function, thereby helping skeletal muscle meet increased energy demands and potentially improve endurance capacity (Wang et al., 2021; Ji and Yeo, 2022). In addition, AMP acts as a signal of energetic stress and can activate AMPK, which may indirectly modulate SIRT1 activity and thereby influence the dynamics of Notch signaling rather than simply its activation state (Ji and Yeo, 2022). Collectively, these mechanisms may contribute to cellular metabolic adaptation, while SIRT1-mediated deacetylation of NICD may fine-tune or even attenuate canonical Notch signaling output depending on the cellular context (Guarani et al., 2011; Bai et al., 2018; Ji and Yeo, 2022).

## 5 Notch signaling in exercise-driven neural adaptation, and cognitive function

**Figure 5:**
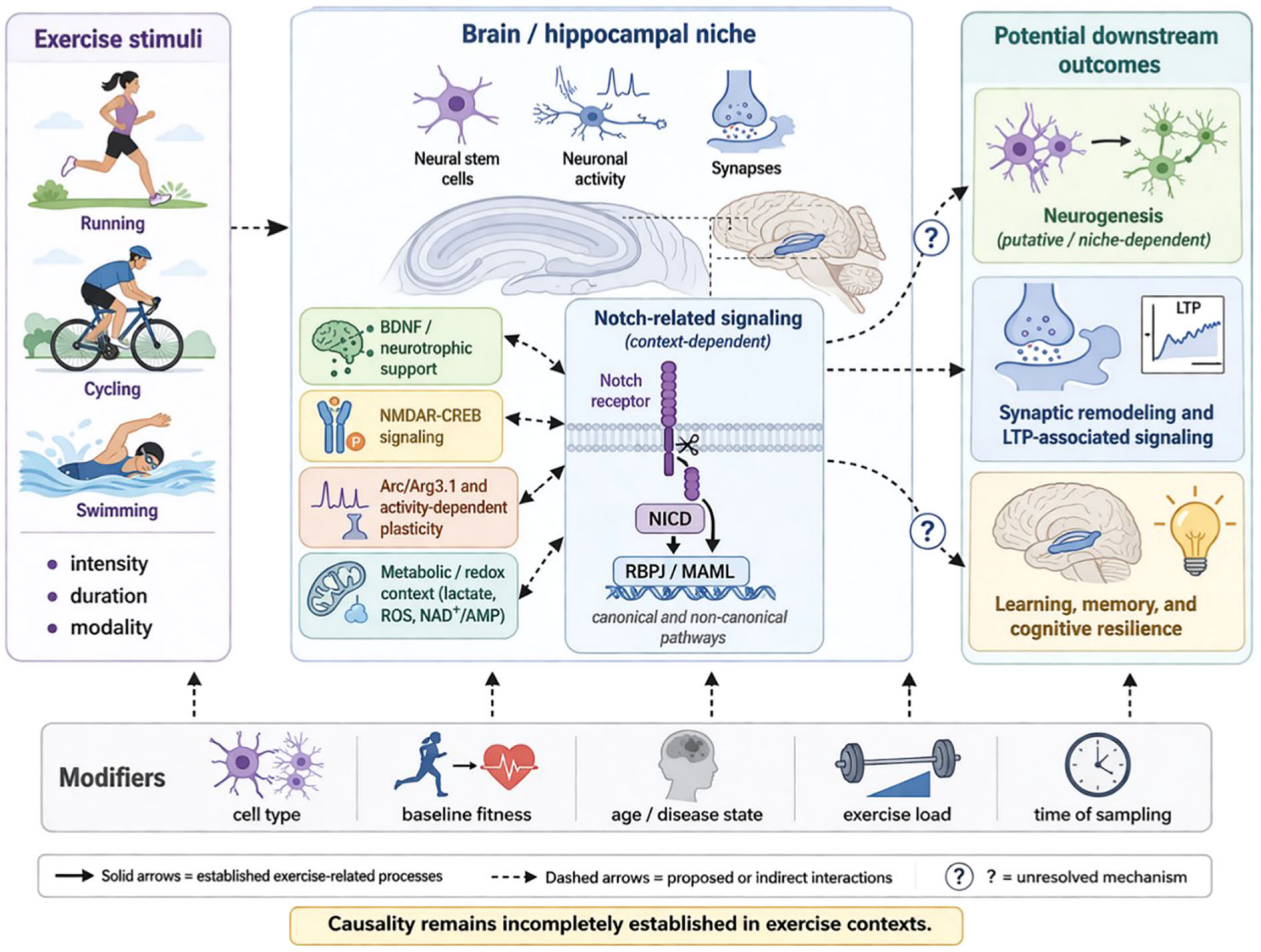
Proposed and context-dependent roles of Notch-related signaling in exercise-associated neural plasticity and cognitive function. This figure presents a context-dependent framework for the potential involvement of Notch-related signaling in exercise-associated neural plasticity and cognitive function. Exercise stimuli, including running, cycling, and swimming, and characterized by intensity, duration, and modality, are known to influence hippocampal neurogenesis, synaptic activity, and neuronal network remodeling through multiple established pathways. Within this framework, Notch-related signaling may interact with neurotrophic support systems such as BDNF, activity-dependent signaling pathways such as NMDAR–CREB, plasticity-associated regulators such as Arc/Arg3.1, and metabolic and redox-related factors including lactate, ROS, and NAD^+^/AMP. However, the extent to which Notch signaling is causally required for these processes remains incompletely understood. Potential downstream outcomes, including neurogenesis, synaptic remodeling, long-term potentiation (LTP)-associated signaling, and improvements in learning, memory, and cognitive resilience, should therefore be regarded as putative and context-specific rather than as universally Notch-driven effects. These relationships are further shaped by multiple modifiers, including cell type, baseline fitness, age or disease status, exercise load, and the timing of measurement. Overall, the figure emphasizes that Notch signaling should be viewed as a context-dependent and potentially modulatory component within a broader multipathway network underlying exercise-induced neural adaptation, rather than as a singular or dominant regulatory mechanism.

By modulating Notch signaling, exercise may promote neuronal renewal, synaptic connectivity, and the efficiency of neural information processing, thereby contributing to learning and memory. The following chapters will examine in greater detail the effects of exercise on neuroplasticity, learning, and memory, as well as the potential role of Notch signaling in these processes (**Figure 5**).

### 5.1 The effect of exercise on neuroplasticity, and learning, and memory

The beneficial effects of exercise on neuroplasticity have been demonstrated in a range of experimental models, and Notch signaling may contribute to the regulation of exercise-associated synaptic plasticity and LTP:

#### 5.1.1 Synaptic plasticity

Exercise enhances synaptic plasticity in the hippocampus and cortex, thereby improving the stability and functional connectivity of neural networks through multiple molecular and cellular mechanisms. Previous studies suggest that exercise may be associated with activation of Notch-related signaling, accompanied by increased synaptic connectivity and hippocampal neurogenesis, both of which are relevant to learning and memory. Notch signaling has been implicated in the regulation of synapse formation and plasticity through downstream transcriptional mediators such as HES1, potentially influencing the efficiency of neuronal information transfer (Liu and Nusslock, 2018; Li et al., 2024).

Within the Notch pathway, HES1 functions as an important downstream effector that may regulate pre- and postsynaptic molecular organization, thereby supporting information transfer and functional connectivity in neural networks. In addition, some evidence suggests that the Notch-HES1 axis may be associated with the promotion of synaptic LTP, which is closely linked to cognitive processes (Liu and Nusslock, 2018).Exercise-associated neuroplasticity is also supported by other pathways, particularly the upregulation of BDNF, a key mediator of hippocampal neurogenesis. BDNF promotes neuronal survival and differentiation and enhances synaptic density and connectivity; accordingly, interactions between BDNF-related and Notch-related pathways may contribute to cognitive adaptation (Li et al., 2024). Together, these findings indicate that exercise may regulate brain function through a complex molecular network that extends beyond its general physiological benefits.

#### 5.1.2 Long-term potentiation

Notch signaling may also participate in exercise-associated LTP in the hippocampus. Previous studies have implicated Notch signaling in memory encoding and consolidation. Notch1, an important cell-communication receptor, has been associated with the regulation of hippocampal synaptic plasticity, partly through interactions with N-methyl-D-aspartate receptor (NMDAR) expression and CREB-related signaling pathways (Brai et al., 2015). Notch signaling may also be enhanced by neuronal activity, and stimuli such as exercise and sensory input have been reported to coincide with activation of Notch-related pathways relevant to synaptic potentiation in CA1 neurons (Alberi et al., 2011; Brai et al., 2015).

In the hippocampus, Notch1 signaling has been reported to interact with molecules such as Arc/Arg3.1, thereby contributing to synaptic structural and functional stability. This interaction may be relevant to exercise-associated LTP, as Arc has been implicated in promoting Notch1 cleavage and strengthening postsynaptic signaling. Such processes may contribute to the long-term maintenance of LTP (Alberi et al., 2011; Brai et al., 2015). Collectively, these findings suggest that Notch-related signaling may support synaptic stability and thereby contribute to long-term memory consolidation and learning. These observations provide a basis for further investigation into the molecular mechanisms through which exercise influences cognitive function.

### 5.2 Notch, and neural network remodeling

Exercise-associated Notch signaling may also contribute to the structural remodeling of neural networks, particularly through effects on synapse formation and connection strength.

#### 5.2.1 Synapse formation

Exercise may influence Notch-related signaling in the brain and thereby support the integration of newly generated neurons and synapse formation through complex multilevel mechanisms. During neurogenesis, Notch signaling regulates the transcription of specific target genes through signaling components such as NICD, CSL, and MAML, including members of the HES and HEY families, which are involved in neural stem-cell fate determination and differentiation (Trinchero et al., 2019; Lampada and Taylor, 2023). Activation of Notch-related pathways may therefore facilitate the integration of newly generated neurons into existing neural circuits, particularly during exercise-associated neurogenesis. Some studies have reported that exercise increases synaptic contacts between newly generated neurons and surrounding cells, thereby enhancing the flexibility of neural networks (Trinchero et al., 2019). In addition, exercise may strengthen synaptic connectivity in newly generated neurons and accelerate their functional integration into neural circuits, thereby increasing network adaptability. Such accelerated integration may improve neuronal signal transmission and promote the formation of more efficient neural circuits, ultimately supporting performance in learning- and memory-related tasks (Lampada and Taylor, 2023).

#### 5.2.2 Connection strength

Notch signaling may also be involved in the regulation of synaptic connectivity and plasticity within neural networks. Previous studies suggest that Notch1-associated signaling may enhance synaptic strength, support the maintenance of LTP, and suppress long-term depression, all of which are synaptic mechanisms relevant to learning and memory. Notch1 has been reported to interact with CREB-related signaling and pathways involving Reelin and NMDARs, thereby potentially improving the precision and responsiveness of information transfer within neural networks (Wang et al., 2004; Brai et al., 2015).

During long-term exercise training, increased Notch-related signaling may further reinforce these adaptive synaptic changes. Some evidence suggests that exercise may enhance Notch-related activity and increase the expression of genes associated with synaptic plasticity, thereby contributing to the stabilization of synaptic strength (Brai et al., 2015). These changes may support the persistence of LTP and improve the overall functional efficiency of neural networks, with possible relevance to cognitive enhancement. Accordingly, Notch signaling may represent one component of the molecular framework underlying exercise-associated neural adaptation and its beneficial effects on cognitive function.

## 6 The potential of Notch signaling in exercise-induced protection against neurodegenerative diseases

### 6.1 Aberrant Notch signaling in neurodegenerative diseases

Notch signaling has been implicated in the pathophysiology of neurodegenerative diseases, although its roles appear to be disease-, cell-type-, and stage-specific. In AD, several studies have reported reduced Notch1-related signaling in the hippocampus and cortex, a pattern associated with synaptic dysfunction, neuronal loss, and cognitive decline (Marathe et al., 2017; Kapoor and Nation, 2021). Because Notch signaling contributes to neuronal differentiation, survival, and synaptic plasticity, its dysregulation may exacerbate neural-network instability and impair learning and memory. In addition, decreased expression of Jagged1, a Notch ligand, has been reported in AD, which may further contribute to deficits in synaptic plasticity relevant to cognition (Marathe et al., 2017).

In Parkinson’s disease (PD), altered Notch-related signaling has been associated with dopaminergic vulnerability, endosomal dysfunction, and neuroinflammation; however, its effects appear to be cell-type-specific rather than uniformly neuroprotective (Imai et al., 2015; Harraz, 2023; Liang et al., 2023); Some studies suggest that impaired Notch signaling may weaken neuroprotective mechanisms and reduce neuronal resistance to oxidative stress and apoptosis, thereby contributing to disease progression (Harraz, 2023). In PD models, reduced Notch expression has been linked to dopaminergic neuronal loss. Notably, myeloid-specific blockade of Notch signaling has been reported to reduce microglia-driven neuroinflammation and alleviate dopaminergic injury, further underscoring the cell-type specificity of Notch effects in PD (Liang et al., 2023). Dysregulated Notch signaling may also contribute to abnormal microglial activation, thereby aggravating neuroinflammation and secondary injury to dopaminergic neurons (Imai et al., 2015; Liang et al., 2023).Accordingly, the role of Notch signaling in PD should be discussed as cell-type- and stage-dependent rather than as a uniformly protective pathway (Imai et al., 2015; Harraz, 2023; Liang et al., 2023).

Overall, dysregulated Notch signaling is associated with pathological changes in both AD and PD, but its disease-specific functions are complex and should not be reduced to a simple loss-of-protection model. In AD, reduced Notch-related signaling may contribute to impaired synaptic plasticity and cognitive dysfunction, whereas in PD, altered Notch signaling appears to influence dopaminergic survival and neuroinflammatory responses in a context-dependent manner. These concepts are summarized in **Table 2**.

**Table 2:** Role of Notch signaling in neurodegenerative diseases: mechanisms, impacts, and therapeutic approaches.

Aspect	Findings	Impact	particular mechanism	References
Aberrant Notch Signaling in AD	Notch signaling is considerably decreased in AD patients’ brains, and particularly, in hippocampal, and cortical neurons	Correlates with neuronal death, synaptic degradation, and cognitive decline	decreased Notch1 activity associates with memory, and learning decline; Jagged1, a Notch ligand, is decreased, impacting synaptic plasticity	(Marathe et al., 2017; Kapoor and Nation, 2021)
Aberrant Notch Signaling in PD	Weakened Notch signaling in PD corresponds with dopaminergic neuron loss	Undermines neuroprotection against oxidative stress, and apoptosis, accelerating disease development	Abnormal Notch signaling activates microglia, heightening neuroinflammation, thus intensifying dopaminergic neuron damage	(Harraz, 2023; Liang et al., 2023; Imai et al., 2015)
Therapeutic Target Potential	Notch signaling functions as a master regulator in adult neurogenesis, and neural stem cell (NSC) maintenance	Dysfunction disrupts neurogenesis, impacting brain resilience	Pharmacological regulation of genes in Notch pathway (e.g., Hes, Hey) can increase neuronal regeneration, and differentiation, possibly alleviating neurodegeneration impacts	(Lampada and Taylor, 2023)
Exercise, and Notch Activation	Exercise activates Notch pathway, increasing neurotrophic factors, and particularly, BDNF (BDNF)	Promotes anti-inflammatory processes, reducing neurodegenerative risks, and supporting neuroprotection	Exercise activates Notch, and decreases inflammation, safeguarding neural function against degenerative changes	(Bonanni et al., 2022; Marques-Aleixo et al., 2021)
Combination Strategy: Exercise + Pharmacology	Combining exercise with pharmacological Notch pathway regulation targets particular neuroprotective benefits	Optimizes neuroprotection by combining natural neuroprotection from exercise, and precise Notch influence via drugs with minimized side impacts	Fine-tuning Hes, and Hey pathways maximizes neuroprotection while reducing side impacts from Notch-targeted therapies	(Lampada and Taylor, 2023)

### 6.2 Potential therapeutic targets

The potential therapeutic relevance of Notch signaling in neurodegenerative disease has received increasing attention. Because Notch signaling contributes to neurogenesis and neural stem-cell maintenance, it represents a biologically plausible target in the central nervous system (Lampada and Taylor, 2023). However, dysregulated Notch signaling is also associated with multiple pathological conditions, including neurodegenerative diseases such as AD and PD, underscoring the need for caution in therapeutic translation (Lampada and Taylor, 2023).

Combining exercise with pharmacological modulation of Notch signaling is conceptually attractive, but at present it should be regarded as a long-term exploratory possibility rather than a near-term therapeutic strategy (Lampada and Taylor, 2023). Exercise has been associated with Notch-related signaling changes and with increased expression of neurotrophic factors such as BDNF, both of which may support neuronal plasticity and anti-inflammatory adaptation (Marques-Aleixo et al., 2021; Bonanni et al., 2022).However, current evidence does not justify the conclusion that exercise exerts its neuroprotective effects primarily through direct activation of Notch signaling.

Pharmacological approaches targeting Notch signaling are also under investigation. Even if future strategies achieve receptor-, ligand-, or cell-type-selective modulation, precise pharmacological regulation of Notch signaling will remain challenging because this pathway is essential for normal homeostasis in the intestine, skin, vasculature, and immune system (Zhou et al., 2022). Any future combination strategy involving exercise and pharmacological modulation would therefore require tissue-selective delivery, receptor- or ligand-specific targeting, and rigorous safety evaluation, because systemic Notch modulation carries substantial gastrointestinal, cutaneous, and immune risks (Wolfe, 2012; Henley et al., 2014; Zhou et al., 2022; Lampada and Taylor, 2023). A major unresolved challenge is how to preserve potentially beneficial Notch-related responses in target tissues while avoiding disruption of Notch-dependent processes in non-target tissues such as the gut, skin, and immune system (Wolfe, 2012; Henley et al., 2014; Zhou et al., 2022).

Overall, exercise-associated and pharmacological modulation of Notch signaling represents a potentially informative multi-mechanistic avenue for future research in neurodegenerative disease. Nevertheless, this concept remains preliminary and should be framed as a hypothesis-generating therapeutic direction rather than an established treatment strategy. **Table 2** summarizes these considerations.

## 7 Integrated model of Notch signaling in exercise-metabolism-neuro system

### 7.1 Proposal of an integrated model

Based on the preceding evidence, we propose an integrated model in which Notch signaling functions as a context-dependent link between exercise, metabolic regulation, and neural adaptation. Within this exercise–metabolism–neural framework, exercise-derived metabolites, such as lactate and ketone bodies, may influence neural plasticity partly through interactions with Notch-related signaling; however, these relationships should currently be regarded as hypothesis-generating rather than as established direct activation pathways (El Hayek et al., 2019; Wu et al., 2023; Plourde et al., 2024). For example, if Notch1 were conditionally deleted in adult hippocampal neural stem cells, exercise-induced neurogenesis would be expected to diminish selectively if Notch were mechanistically required in that niche. Likewise, if lactate directly modulates Notch signaling, perturbation of lactate transport or intracellular pH buffering should alter Notch-reporter activity under exercise-mimetic conditions (Imayoshi et al., 2010; El Hayek et al., 2019; Wu et al., 2023). Future studies should therefore combine conditional knockout models, receptor- or ligand-selective perturbation, reporter systems, and single-cell or spatial approaches to determine when exercise-associated Notch responses are causal, compensatory, or merely correlative (Friedrich et al., 2022; Zhou et al., 2022; Lampada and Taylor, 2023).

This model emphasizes the potential bridging role of Notch signaling between metabolic by-products and neural adaptation. Rather than assuming that exercise-derived metabolites directly activate Notch signaling in a uniform manner, the model proposes that Notch-related responses are shaped by tissue context, cell state, and metabolic conditions. In this view, Notch signaling may participate in the coordination of metabolic and neural processes that support adaptation to physiological and environmental demands. Accordingly, this integrated framework may provide a useful conceptual basis for future studies exploring the role of Notch signaling in metabolic and neurological disorders.

### 7.2 Application value of the model

This model may have several potential applications in the design of exercise interventions, metabolic therapies, and Notch-targeted treatment strategies. First, it may provide a theoretical framework for individualized exercise interventions by helping to interpret how Notch-related responses vary according to metabolic and neurological status. Such an approach could support the development of more tailored exercise programs aimed at improving rehabilitation outcomes. Second, by highlighting interactions between Notch signaling and metabolic pathways, including glucose and lipid metabolism, the model may offer conceptual guidance for therapeutic strategies in metabolic disorders. Third, the model may inform the rational design of Notch-targeted interventions in neurological and metabolic disease contexts. However, these applications remain largely theoretical and will require substantial experimental validation before clinical translation can be considered.

### 7.3 Limitations, contradictory evidence, and experimental priorities

Despite growing interest in the intersection of exercise biology and Notch signaling, the current evidence base remains limited in several important respects. First, much of the literature discussed in this review does not directly examine exercise-induced Notch signaling *in vivo*, but instead derives from developmental biology, disease models, tumor systems, or non-exercise metabolic contexts. Although such studies are mechanistically informative, they should not be interpreted as direct evidence that exercise uniformly activates or suppresses Notch signaling across tissues. In addition, a substantial proportion of the available evidence is correlative rather than causal. In many studies, exercise or exercise-like stimuli are associated with changes in neurogenesis, synaptic plasticity, redox balance, or muscle regeneration, yet direct manipulation of the Notch pathway is not performed. Consequently, it remains unclear whether Notch acts as a primary driver, a permissive modulator, a compensatory response, or simply one component of a broader adaptive network.

Tissue specificity also remains insufficiently resolved. Notch signaling is highly context-dependent and varies according to receptor subtype, ligand availability, cell type, developmental stage, and microenvironmental conditions. Therefore, findings observed in hippocampal neural stem cells, satellite cells, cardiomyocytes, endothelial cells, microglia, or hepatocytes should not be assumed to reflect a shared exercise-response pattern. Direct human evidence is also scarce. Most mechanistic insights currently derive from rodent, cell-culture, or other experimental systems, whereas human exercise studies rarely assess canonical or non-canonical Notch pathway activity with adequate spatial and temporal resolution. This limits translational confidence. Moreover, exercise adaptations are mediated by multiple intersecting pathways, including BDNF, AMPK, mTOR, PI3K/Akt, Wnt, Shh, Nrf2, inflammatory signaling, and mitochondrial quality-control networks. Although Notch may interact with these pathways, current evidence does not justify treating it as a universal or dominant mediator of exercise-induced neuro-metabolic adaptation. Finally, several mechanistic links discussed in this review, particularly those involving lactate, ketone bodies, intracellular pH, NAD+/SIRT1, and multi-organ coordination, should currently be viewed as hypothesis-generating rather than as established linear pathways. Future work should therefore prioritize conditional and cell-type-specific genetic models, receptor- and ligand-selective perturbation, exercise protocols with clearly defined internal-load metrics, time-resolved reporter systems, and single-cell or spatial analyses to determine when Notch signaling is causally required, when it is secondary, and when it is not involved.

### 7.4 Future study directions

Building on this framework, future research should further clarify the mechanisms through which Notch signaling participates in exercise-associated adaptation. One priority is the development of precision strategies for modulating Notch signaling, including gene-editing and pharmacological approaches capable of achieving cell-type- or receptor-specific control. Such strategies may help enable more individualized therapeutic regulation of Notch-related responses. A second priority is the investigation of long-term exercise interventions and their effects on Notch-related signaling in chronic neurological and metabolic disease contexts. Better characterization of the temporal and tissue-specific dynamics of Notch signaling during prolonged exercise may help establish a stronger theoretical basis for the long-term benefits of exercise-based interventions and for their potential use as adjunctive approaches in neurodegenerative disease management.

## 8 Conclusion

Notch signaling is a biologically important pathway involved in stem-cell regulation, tissue homeostasis, neural plasticity-associated processes, and selected aspects of metabolic control. These established functions make it a plausible candidate for participation in exercise-responsive adaptation. However, current evidence does not support a universal model in which exercise directly and consistently activates or suppresses Notch signaling across tissues. The strongest exercise-relevant mechanistic evidence currently relates to neural stem-cell biology, synaptic plasticity-associated signaling, and skeletal-muscle stem-cell regulation. By contrast, many proposed links involving hepatic metabolism, metabolite-mediated activation, and system-level neuro-metabolic coordination remain indirect or hypothesis-generating. Exercise-associated Notch responses should therefore be interpreted as context-dependent and likely to vary according to tissue type, cell type, receptor-ligand composition, internal load, redox status, and baseline physiological state. Overall, Notch signaling should be regarded not as an established master regulator of exercise adaptation, but as a plausible and testable component of a broader multipathway response network. At present, its greatest value lies in informing future mechanistic investigations rather than immediate clinical translation.
