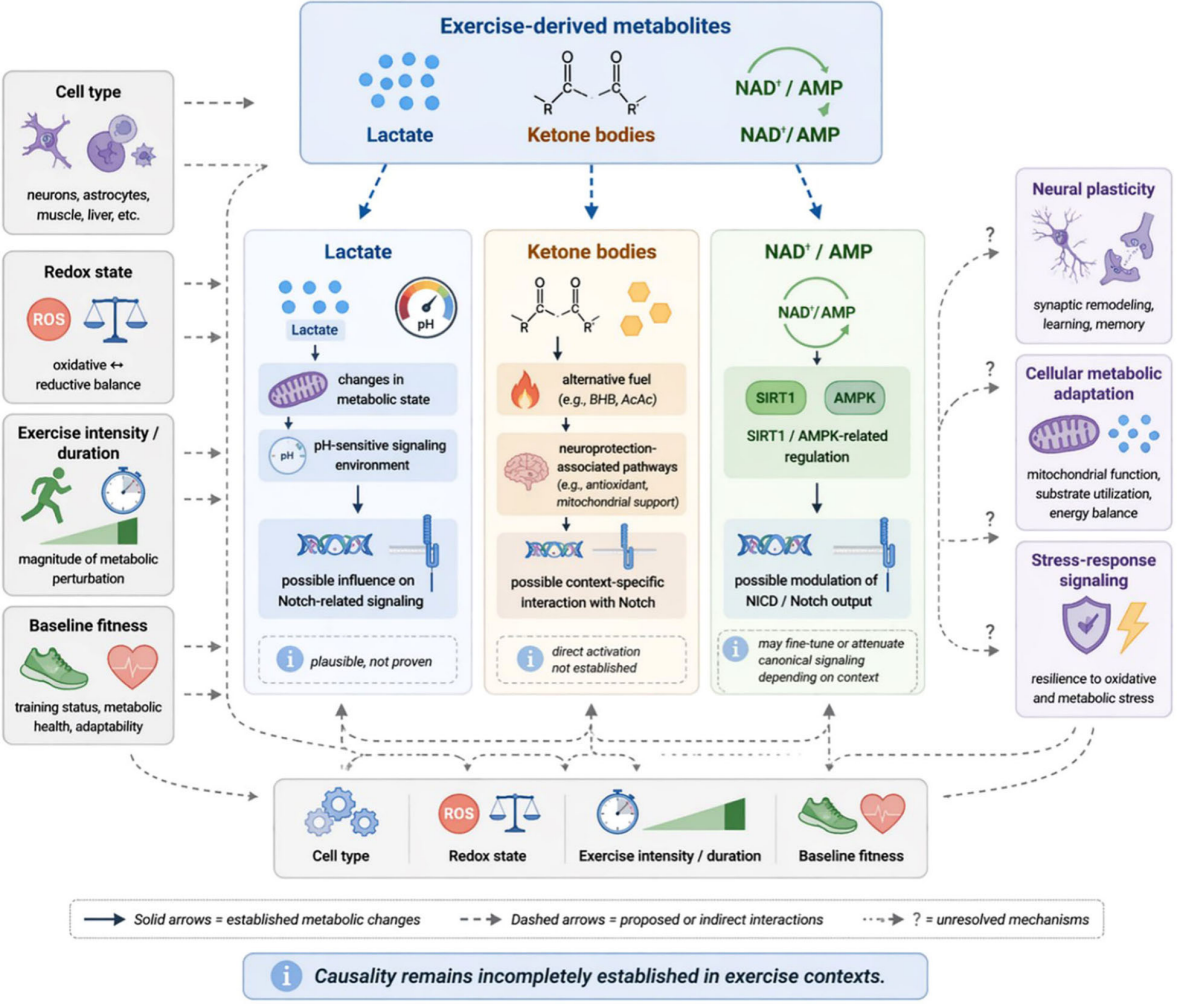# Correction: What role does the Notch signaling pathway play in exercise-related metabolic and neurological adaptations? A molecular-to-systems perspective

**DOI:** 10.3389/fphys.2026.1908131

**Published:** 2026-06-19

**Authors:** Lin Li, Jianda Kong, Xuewen Tian

**Affiliations:** 1 Department of Sports Science Research Institute, Shandong Sport University, Jinan, China; 2 Department of Physical Education, Qufu Normal University, Jining, China

**Keywords:** exercise, metabolic homeostasis, neurogenesis, neuroplasticity, Notch signaling, oxidative stress

The figures were in the wrong order in the PDF version of this paper. In the initial PDF file ofthis paper, **Figure 1** and **Figure 2** were placed in the wrong sequence. We have revised their order, and the latest version of the article has been updated. The order has now been corrected.

The original version of this article has been updated.

**Figure 1:**
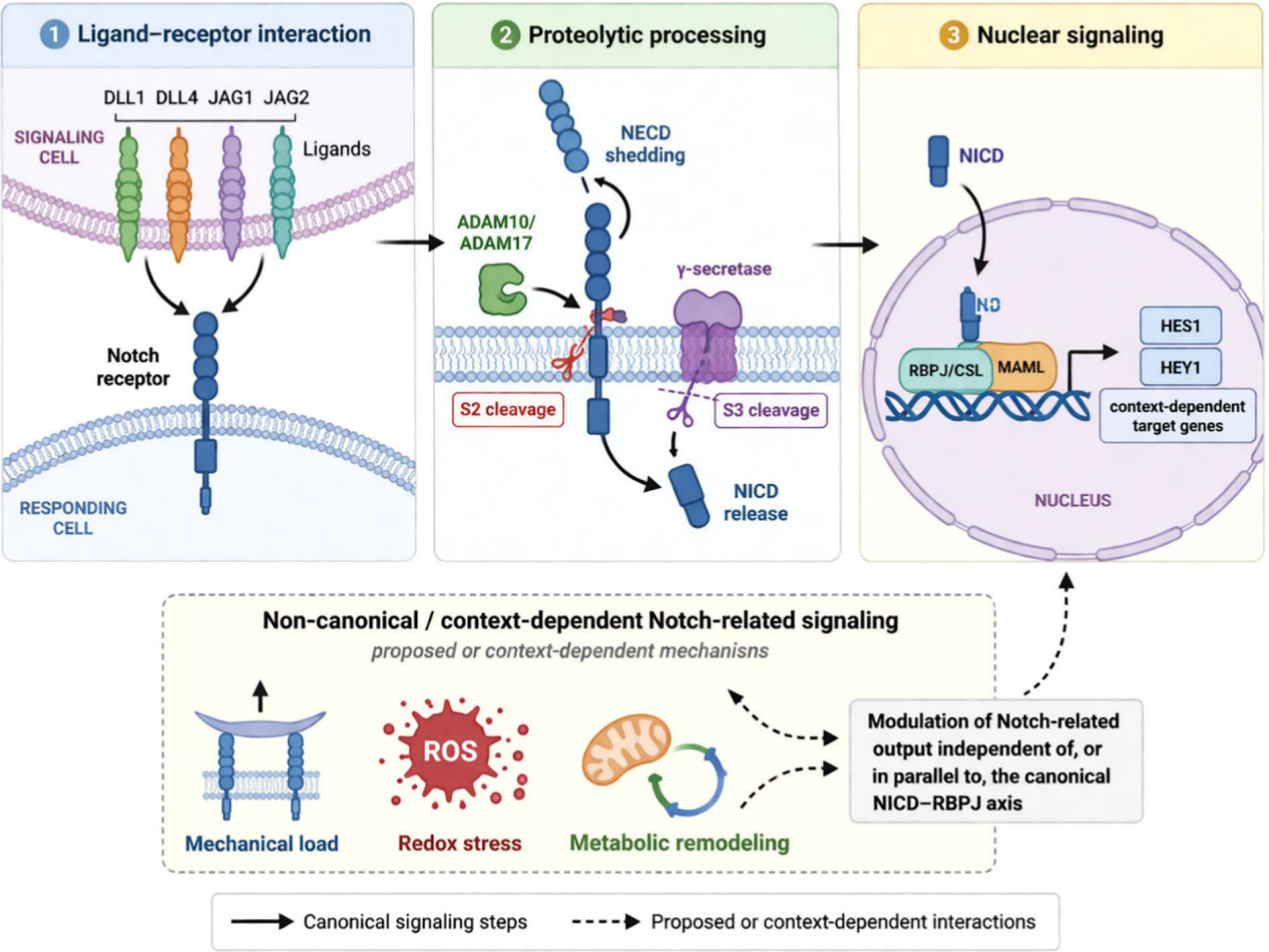


**Figure 2:**